# A Preliminary Exploration of Pitch Discrimination, Temporal Sequencing, and Prosodic Awareness Skills of Children Who Participate in Different School-Based Music Curricula

**DOI:** 10.3390/brainsci11080982

**Published:** 2021-07-24

**Authors:** Ashley G. Flagge, Mary Ellen Neeley, Tara M. Davis, Victoria S. Henbest

**Affiliations:** Department of Speech Pathology and Audiology, University of South Alabama, Mobile, AL 36688, USA; men1821@jagmail.southalabama.edu (M.E.N.); taradavis@southalabama.edu (T.M.D.); vhenbest@southalabama.edu (V.S.H.)

**Keywords:** pitch discrimination, auditory processing, prosodic awareness, music education

## Abstract

Musical training has been shown to have a positive influence on a variety of skills, including auditory-based tasks and nonmusical cognitive and executive functioning tasks; however, because previous investigations have yielded mixed results regarding the relationship between musical training and these skills, the purpose of this study was to examine and compare the auditory processing skills of children who receive focused, daily musical training with those with more limited, generalized musical training. Sixteen typically developing children (second–fourth grade) from two different schools receiving different music curricula were assessed on measures of pitch discrimination, temporal sequencing, and prosodic awareness. The results indicated significantly better scores in pitch discrimination abilities for the children receiving daily, focused musical training (School 1) compared to students attending music class only once per week, utilizing a more generalized elementary school music curriculum (School 2). The findings suggest that more in-depth and frequent musical training may be associated with better pitch discrimination abilities in children. This finding is important given that the ability to discriminate pitch has been linked to improved phonological processing skills, an important skill for developing spoken language and literacy. Future investigations are needed to determine whether the null findings for temporal sequencing and prosodic awareness can be replicated or may be different for various grades and tasks for measuring these abilities.

## 1. Introduction

Musical training has been shown to have a positive influence on a variety of skills and abilities, including auditory-based tasks and nonmusical cognitive and executive functioning abilities, such as inhibition, working memory, and general cognition [1]. Additionally, musical training impacts an individual far beyond childhood and even into later adulthood [2], and research suggests that musical training beginning in childhood has potentially more significant positive long-term effects than when begun in adolescence or adulthood [3]. Because of this, many studies have been geared toward investigating the use of musical training in educational settings [4]. Because auditory processing skills have been found to be important for speech and language development, including literacy skills, previous research has focused on the relation and effects of early musical training on auditory processing [5,6,7]. While there is some disagreement in the literature regarding the influential extent of musical training on phonological processing and reading, the general consensus is that musical training in childhood does lead to at least some degree of improvement in phonological awareness and other auditory processing skills (see [8] for a review).

One area of auditory processing that has been shown to be related to musical experience is pitch processing [9,10]. Pitch processing ability is often assessed with the use of a discrimination task, in which an individual is asked to report on perceived differences in pitch. In educational settings, better pitch discrimination skills have been associated with better phonological processing and reading skills [10], and regression analysis has shown that pitch discrimination thresholds in adults predict phonological awareness beyond what is predicted by phonological short-term memory and rhythm discrimination [11]. Musical training has often been shown to contribute to pitch discrimination abilities in both adults [12,13,14,15,16] and children [3,10,17]. Prior research in adult populations has found that adults with a history of both vocal and instrumental musical training show better pitch discrimination ability compared to adults with no significant musical training, although the length of musical training varies considerably among studies [10,12,13,14,15]. While there is some debate on the amount of musical training needed to influence pitch discrimination thresholds [15], Smith et al. [17] found that formal musical training beginning prior to age six had the most significant impact on pitch discrimination ability in adults after controlling for sex, age, native language, and general intelligence. Hutka et al. [14] found that musicianship in adults also shows benefits for speech processing and sensitivity to timbral characteristics of speech, suggesting carryover effects of pitch discrimination into the area of speech processing.

In addition to pitch, incoming auditory information is also processed in the temporal domain. Temporal processing is the ability to perceive changes in acoustic stimuli across time, such as the perception of short gaps of silence between phrases or during the production of stop consonants. Temporal sequencing refers to an individual’s ability to sequence a pattern of auditory events over time [18]. Temporal sequencing ability has been found to be crucial in processing several aspects of language, as well as music [19,20,21]. Music training has been associated with better auditory temporal processing in several domains, including temporal resolution and sequencing, suggesting potential improvement specifically in the temporal sequencing domain [5,6,9]. Because temporal sequencing has been linked to improved language and reading abilities [9,22,23], the finding of improved sequencing outcomes following musical training in childhood holds important educational curriculum implications.

Prosodic awareness studies have also been conducted in relation to musical training and improved reading abilities [17]. Prosody can be defined as the “rhythmic patterning of speech,” such as intonation, stress, and spacing. Prosodic awareness refers to the ability to consciously think about these rhythmic patterns [24]. Importantly, prosodic processing incorporates elements of both spectral (pitch) and temporal processing, and it is important in the comprehension of meaning in both language and music. For example, prosodic processing allows for the discrimination of questions and statements in speech and allows for the processing of a melodic contour in music. Research has shown that prosodic awareness skills are related to better educational outcomes, including reading [25,26,27,28]. However, research examining the effects of musical training on prosody, specifically prosodic awareness, or the rhythmic patterning of speech, show conflicting results. Some studies have shown relations between receptive and expressive prosodic processing in both adults and children and improved prosodic processing following musical training [29,30]. Other studies, however, have correlated prosodic awareness to other cognitive aspects not related to musical training, such as emotional intelligence [31].

While there is an abundance of research showing positive correlations between music training and pitch processing, the research focused on the relation between musical training and the domains of temporal sequencing and prosodic awareness is sparser, particularly in children. Therefore, the present study was conducted to examine whether two groups of children from two elementary schools who received different school-based music curricula would perform differently in three aspects of processing: pitch discrimination, temporal sequencing, and prosodic awareness. The students from the schools (School 1 and School 2) were differentiated based on both the amount of musical training received as a part of the school curriculum and the specific music curriculum used. The following research questions were proposed:

1. Do children who receive focused daily musical training show better behavioral pitch discrimination thresholds compared to children receiving more generalized music training once per week?
2. Do children who receive focused daily musical training show a difference in temporal sequencing compared to children receiving more generalized music training once per week?
3. Do children who receive focused daily musical training show a difference in prosodic awareness compared to children receiving more generalized music training once per week?

Based on prior research, it was expected that children who receive the focused daily musical training curriculum would show better (lower) pitch discrimination thresholds [3,10,17] and better (higher) scores on both temporal sequencing [5,6] and prosodic awareness tasks [29,32] compared to students who receive music only once per week.

## 2. Materials and Methods

### 2.1. Participants

Sixteen children (eight from each school) were recruited for participation through two private schools in the southeastern United States. Students at School 1 attended a music class every day as part of their curriculum. Students at School 2 attended one 45-minute music class per week. To recruit study participants, information on the study was sent home to all children in second–fifth grade at both schools. Parents who were interested in having their child participate completed IRB-approved consent forms and questionnaires regarding medical history and musical experience. Children with any reported diagnosis of neurological, language, or reading disorder were excluded from the study. None of the children reported a significant external musical history, defined as greater than three years of individual vocal or instrumental lessons. All children passed a bilateral pure tone hearing screening (octave frequencies between 250–4000 Hz) prior to participation. All children were reported to be either A or A/B honor roll students.

### 2.2. Music Curriculum

School 1 used the Abeka academic curriculum with supplemental materials based on individual ideas and research. The music curriculum was focused and specific to each student’s age and skills, utilizing a combination of lessons based on developmental age and current class curriculum. Students received a traditional education in music theory, sight reading, ear training, and vocal and instrumental technique. Instruments included the ukulele, traditional piano, and traditional Orff technique on xylophones and drums. Younger students performed tasks such as differentiating pitch by identifying “high or low”, tempo by “fast or slow”, and dynamics by “loud or soft”. As students developed musical skills, pitch relations for major scales and chord building were also incorporated. All students recruited from School 1 had attended the school since kindergarten and received daily musical instruction for the duration of the school year.

School 2 was a Southern Association of Colleges and Schools (SACS) accredited Catholic elementary and middle school. Students in the elementary school all attended a music class once per week for 45 min. During this time, students engaged in group singing and played basic percussive instruments. While this curriculum did engage students beyond passive music exposure (i.e., listening to music), it did not provide specific pitch interval or instrumental training. Because students only received a generalized music curriculum once per week rather than a daily class with a specific and focused training curriculum, these students acted as the comparison group, and were matched to the children from School 1 by both age and parental education level. None of the students attending School 2 received any musical training outside of the once per week class provided by the school.

Group matching was based on both age and parental education level as an indicator of socioeconomic status [33]. To analyze parental education level, the levels were categorized into four groups and given numerical values: high school/GED (1), some college/trade school/associate’s degree (2), bachelor’s degree (3), and graduate level degree (4). Statistical analysis (*t*-test) revealed no significant difference between the ages of the children (*p* = 0.982) in School 1 (mean age (MA) in months: 100.75; standard deviation (SD): 11.63) and School 2 (MA: 100.63; SD: 10.64). There was also no significant difference in the parental educational level (*p* = 0.266) of School 1 (M: 2.31; SD: 0.75) and School 2 (M: 2.81; SD: 0.96). Socioeconomic status (SES) was used to match the groups, as there is evidence that children from various SES backgrounds can have different linguistic and academic outcomes [34]. An independent sample *t*-test was also completed to examine the years of musical training between the groups, as considered from case history information provided by parents. Since no students from School 2 were involved in any extracurricular music training, the number of years of training was based on their involvement in the one music class per week that was completed through the school curriculum. Students from School 1 also reported no additional musical training prior to the start of kindergarten, with the exception of one student, who was involved in church choir ensembles since the age of two years. Analysis of musical training revealed no significant differences in mean years of training between School 1 (M: 3.25 years; SD: 1.04) and School 2 (M: 3.00 years; SD: 0.76).

### 2.3. Tasks

Pitch discrimination was assessed using an adaptive psychophysical assessment protocol that yielded a difference limen for frequency (DLF) for each child. Stimuli were created and presented using the Psychoacoustics toolbox [35] in MATLAB. Children were asked to complete a four-interval, two-alternative forced choice forward task (4I-2AFC), in which four tones were divided into two groups. The children were asked to determine which group (1 vs. 2) had the “different” sound. Stimuli were presented using a parameter estimation by sequential testing (PEST) protocol with a *W* constant of 1 and a *p*-target of 0.8 (80%). The standard reference tone was presented at 220 Hz, with the starting comparison tone presented at 320 Hz. The initial step size was 50 Hz, and a final step size of 0.5 Hz was programmed into the software. The comparison tone minimum was 220 Hz, so that the “different” sound was always presented at a higher frequency than the standard 220 Hz tone. The comparison tone was also always presented as the second tone of the pair, so that the standard 220 Hz tone was always presented as the first tone in each group of tones. Per the adaptive protocol, the frequency difference became increasingly smaller with correct responses, and the total number of trials was individualized for each participant. The PEST algorithm determined the final DLF, with smaller numbers indicating better pitch discrimination. This 4I-2AFC forward protocol was chosen due to prior research [36] and unpublished pilot data suggesting that this protocol elicits the best pitch discrimination scores in children.

For this task, the children were asked to determine which grouping of tones (1 or 2) contained the “different” sound. They were told to indicate their choice by pointing to a visual representation of 1 vs. 2 contained in a square block. Holding up one or two fingers was also accepted as a response. Many children also verbally responded, and this was allowed. There were no noted discrepancies between the verbal and physical response. The children were given a short practice block of five trials to ensure task understanding. If they were unable to complete the task independently after five practice trials, they were allowed five more practice trials. If they were still unable to complete the task, they were disqualified from the study.

The *Pitch Pattern Test (Musiek Version)* is a test of auditory temporal sequencing. For each trial, three tones are presented sequentially with a frequency of either 880 Hz or 1122 Hz. Each tone is 200 ms in duration, with 150 ms of silence between each tone. Listeners are asked to correctly order the tones by verbally labeling them as “high” or “low” (e.g., “high, high, low”). There are six possible answer combinations: LLH, LHL, LHH, HLH, HLL, and HHL [37].

The *Duration Pattern Test (Musiek Version)* is also considered a test of auditory temporal sequencing, in which listeners are asked to order three sequentially presented tones according to duration, rather than frequency. Each 1000 Hz tone presented is either 500 ms or 250 ms in duration, with 300 ms of silence between each tone. Listeners respond by verbally labeling each tone as either “long” or “short”. There are six possible combinations: LLS, LSL, LSS, SLS, SLL, and SSL [37].

The PPT and DPT were presented using a full presentation list (30 items for each test), and a total number of correct responses was recorded. The children were asked to linguistically label the order of the tones by use of either high and low (for PPT) or long and short (for DPT). Any error in the response for any of the three stimuli per trial resulted in an incorrect response.

The *Profiling Elements of Prosody in Speech Communication 2015 (PEPS-C)* [38] is a test of receptive and expressive prosodic ability in both adults and children. The interest in the present study was primarily in assessing prosodic awareness, rather than production, and, therefore, only receptive tasks were utilized. The prosodic function sections used in the study included tasks for turn end, affect, boundary, and contrastive stress. Each subtest included three practice trials to ensure understanding of the task, followed immediately by 16 experimental items. Turn end tasks required the participant to determine whether the female speaker was asking a question or making a statement (for example “Carrots?” or “Carrots.”). Affect tasks determined whether the speaker liked what they were talking about or had reservations, such as “carrots” (sounding happy) or “carrots” (sounding unhappy). Boundary tasks used prosodic phrase boundaries to determine “chunking” (fruit, salad, and milk, or fruit salad and milk). Contrastive stress tasks emphasized a specific word in an utterance (“I wanted blue and BLACK socks” vs. “I wanted BLUE and black socks”).

For each task, the children were presented with two different auditory stimuli and images on the computer screen. They were given brief instructions and three practice trials prior to beginning each task. The children were asked to indicate the correct image that matched the auditory stimulus presented by pointing to the image. Some children responded verbally as well, but scoring was based on whether the child pointed to the correct image, and no discrepancies between verbal and motor responses were noted. The examiner clicked the correct response on the computer screen, and the student’s score for each subtest was saved within a spreadsheet in the *PEPS-C* software.

### 2.4. Procedure

The children were assessed in one session in a quiet room at each respective school. After signing a consent form and completing a hearing screening, children were instructed on each task. All tasks were presented via headphones connected to a laptop computer. The volume of the computer was set to 50%, and all tasks were presented binaurally. All children reported being able to hear the stimuli easily and comfortably for all tasks. Binaural presentation was chosen due to prior research suggesting that there are no significant performance differences in auditory sequencing performance between the ears of presentation [39,40]. All tasks were counterbalanced.

## 3. Results

### 3.1. Descriptive Statistics

Table 1 shows the scores for each of the seven tasks for each participant. The DLF scores are reported in Hz, with lower reported scores indicating smaller DLF thresholds and suggesting better pitch discrimination. All other measures are reported in percent correct scores.

A visual comparison of mean scores for each group revealed lower DLF scores (indicating better performance) and higher percent correct scores for the students from School 1 for all test measures, except the Boundary subtest in the *PEPS-C* (Table 2).

### 3.2. Analytical Statistics

To address the three research questions examining whether children who receive different music curricula demonstrate differing pitch discrimination ability, temporal sequencing, and prosodic awareness, parametric statistics were used for each measure when the data met the assumptions. For measures in which the assumptions were not met, the non-parametric alternative was conducted, and the assumption that was violated was stated. Because of the small sample sizes, the effect sizes measured as eta squared (Mann–Whitney U) or partial eta squared (ANOVA) were the focus of the analyses.

**Figure 1:**
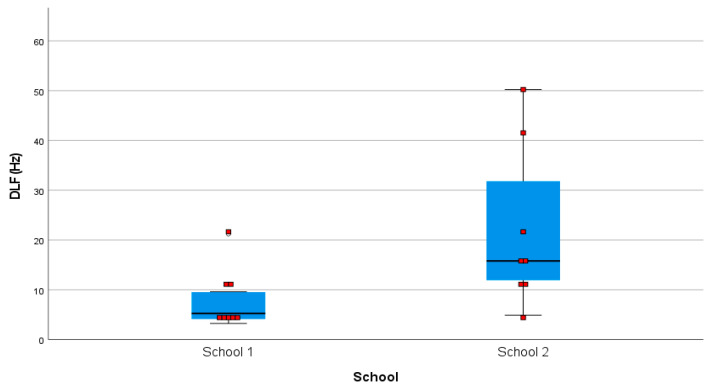
Mean pitch discrimination scores (DLF) showing variance for each group.

To examine whether there was a difference between groups in pitch discrimination ability, the DLF scores were compared. The Shapiro–Wilk test of normality was significant (*p* = 0.009) for DLF, suggesting a violation of the normality assumption; therefore, the Mann–Whitney U test for nonparametric data was utilized. A two-tailed Mann–Whitney U test revealed significant differences between groups (*p* = 0.015), indicating that the children from School 1 who received focused, daily musical training had significantly better pitch discrimination scores with less variance compared to the children from School 2 (Figure 1). Importantly, the effect size for the difference in performance between the two groups was very large (η^2^ = 0.37), indicating that this finding was of practical importance. 

**Figure 2:**
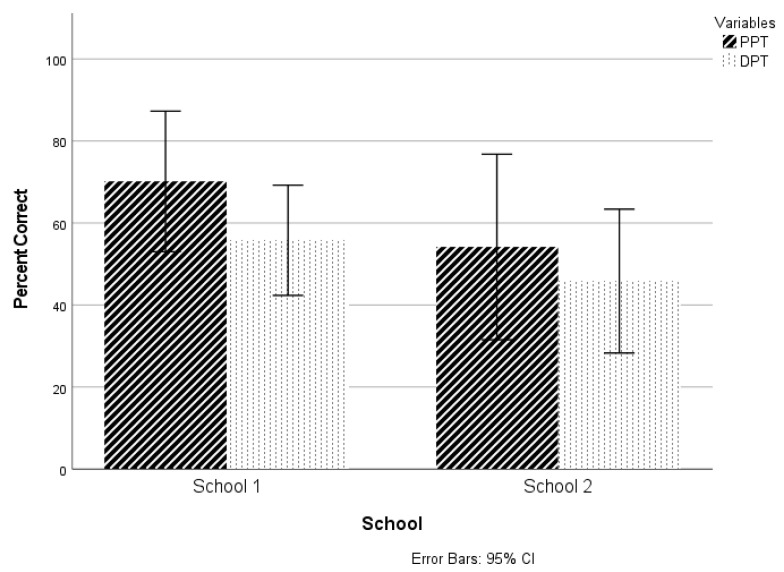
Mean percent correct scores and error bars (95% CI) for temporal sequencing tasks (PPT and DPT for each group).

To examine whether children who received different musical curricula demonstrated different temporal sequencing ability, a two-way (2 × 2) mixed model ANOVA was completed with the group as one factor and test (PPT vs. DPT) as the second factor. The results revealed no significant interaction effects (F(1,14) = 0.247, *p* = 0.627, η*p*^2^ = 0.017) or main effects of group (F(1,14) = 2.131, *p* = 0.166, η*p*^2^ = 0.13) or test (F(1,14) = 3.499, *p* = 0.082, η*p*^2^ = 0.2), indicating that: (1) children who received focused, daily musical training did not perform significantly better on temporal sequencing measures compared to children who did not receive focused, daily musical training, and (2) scores on the PPT and DPT were not significantly different from each other (Figure 2). It should be noted, however, that scores for the children who received the more specific, daily musical training were descriptively 16 percentage points higher for the PPT and 10 percentage points higher for the DPT than children from School 2. Also of note is that the effect size for both group and test comparisons was large (η*p*^2^ = 0.13, 0.2, respectively), suggesting that children who received more focused and frequent musical training did in fact demonstrate more robust temporal sequencing skills compared to children attending music class only once per week, and that children who received more focused and frequent training performed better on the PPT compared to the DPT.

To examine whether children who received focused, daily musical training differed from children who did not regarding their prosodic awareness skills, a two-way (2 × 4) mixed ANOVA was completed with group as one factor and subtest as the second factor. The results revealed no significant interaction effects or main effect for group (F(1,14) = 0.558, *p* = 0.468, η*p*^2^ = 0.038), indicating that there were no significant differences in prosodic awareness performance between children from School 1 and School 2. The results did reveal significant main effects for the subtest with a large effect size (F(3,42) = 9.67, *p* < 0.001, η*p*^2^ = 0.408), suggesting that the type of prosodic awareness task was a significant contributor to performance. Post hoc testing (LSD) revealed that the turn-end subtest had significantly higher scores than the remaining three subtests.

## 4. Discussion

A statistical analysis of the children’s performance revealed a significant difference in pitch discrimination abilities between groups, but no significant differences in temporal sequencing or prosodic awareness. The results examining pitch discrimination confirmed our hypothesis that children who received focused, daily musical training (School 1) performed significantly better on a pitch discrimination task than children who received more generalized musical training once per week (School 2). Prior research has consistently shown a strong correlation between pitch discrimination abilities and musical training in children, even with less frequent training, compared to the children in the present study [10,17]. The present study confirms these findings, indicating that students who receive focused, daily music training have better pitch discrimination than children who participate in more general and less frequent musical training. In comparison to prior studies using the same 4I adaptive discrimination methodology in similarly aged children (Sutcliffe & Bishop, 2005), it was found that the students in both groups in the present study showed better mean and standard deviations, although the students from School 1 showed much better means and much less variation compared to School 2. However, it should be noted that, within both groups, there was still a range of DLF scores (Table 1), which were not significantly related to years of musical training within each school. This speaks to the multifaceted nature of pitch discrimination ability, indicating that, while musical training certainly seems to play a role, there are likely other contributing factors at play also.

Analysis of temporal sequencing tasks revealed no significant differences between groups on either measure of temporal sequencing. It should be noted that, unlike pitch discrimination in School 1, there was no specific curricular activity in either school designed to target temporal sequencing. Importantly, however, there was a trend towards better performance in both the PPT and DPT for the children who received daily musical training. Despite the lack of statistical significance, the large effect size for group suggests the need for further investigation into the effects of daily musical training on temporal sequencing ability in children.

Finally, analysis of prosodic awareness revealed no significant differences between the two groups of students. Again, it should be noted that there was no specific activity in either school’s music curriculum to target prosodic awareness. However, in examining the findings more closely, it was noted that the contrastive stress subtest, in which the participant listened for an emphasized word in an utterance, revealed a higher mean score of 10 percentage points for School 1 versus School 2, a difference between groups more than double the size of differences for any other prosodic subtest (Table 2). While the acoustic contributions to contrastive stress in English are debated, many agree that frequency (F0) is the primary cue used by listeners compared to duration and intensity (see [41] for a review). It is therefore possible that the better pitch discrimination performance observed in students from School 1 could also be related to the better contrastive stress scores in these students. However, further research is needed to confirm this finding.

Despite the small sample size, significant differences and large effect sizes were still found in pitch discrimination abilities between School 1 and School 2. Because the students in the two schools received different frequency of training (daily vs. weekly) and different curricula (focused, specific pitch and instrumental training vs. a more generalized group singing and percussive instrument playing), it is difficult to disentangle the effects of the frequency of training and focus of the curriculum. It is possible that the more focused pitch training conducted in School 1 played a role in the better outcomes observed. It is also possible that the frequency of training (daily for 5 h/week compared to weekly for 45 min/week) was at play. More likely, however, is that a combination of these two factors is important for pitch discrimination. While further research is needed to directly examine the contributions of both frequency and type of training in this age group of children, these preliminary results speak to the potential impact and importance of the use of focused and frequent musical training as part of the elementary curriculum.

Overall, differences were noted in more basic skills, such as pitch discrimination, but not necessarily in more complex, higher-level tasks. These findings support previous research in pediatrics, suggesting that higher level processing skills take more training time to show an improvement in scores [3,9]. Pitch discrimination is considered a lower-level auditory skill [42], and therefore may be an earlier developing skill, following musical training [3]. However, higher-level skills, such as temporal sequencing abilities and prosodic awareness, are hypothesized to require more years of musical training and experience to show improvement [3]. Therefore, it is possible that the students in the present study had not experienced musical training for a long enough duration to produce significant group differences in more complex, higher-level auditory processing abilities. Another possibility for the lack of statistically significant differences in temporal sequencing and prosodic awareness tasks is that, as mentioned previously, neither temporal sequencing nor prosodic awareness were explicitly taught in either school curriculum. Pitch discrimination and interval training, however, were targeted skills in School 1′s curriculum. Therefore, it is possible that the specific and targeted pitch training in the curriculum for School 1 contributed to the better pitch discrimination scores observed in these students.

Finally, it is possible, based on our findings, that temporal processing and prosodic awareness skills are not associated with the amount and type of musical training, as no statistically significant differences were found in the performance of these tasks between the two schools. However, this interpretation seems unlikely for several reasons. First, prior research examining the effects of musical training have shown better outcomes, especially on temporal sequencing [5,6,9], compared to those not receiving training. Second, our data showed descriptive trends towards better outcomes for both temporal sequencing and several prosodic awareness subtests (especially contrastive stress) for students from School 1. Finally, the large effect sizes seen in our data indicate that these findings do have practical significance and should be further investigated with larger samples to determine the impact of musical training on each of these processes.

Another potential limitation to the present study is that no language, memory, or cognitive pretesting was performed, all of which potentially play a role in performance [43]. While all students performed similarly academically (all A or A/B honor roll), there was no objective pretest measurement obtained prior to the start of the study. Additionally, no baseline pitch discrimination, temporal sequencing, or prosodic awareness measures were obtained from students from either school prior to beginning the school music curriculum. Because both schools were private schools, there is a possibility that parents chose the school with a more focused music curriculum because they noted a predisposition to music in their child. Future studies should seek to confirm these findings in larger and more diverse samples of typically developing children, and examine the effects of daily music training provided through the school curriculum on other auditory processing, language, and literacy abilities. Additionally, further research could aim to explore the effects of focused, daily musical training on clinical populations. It is known that individuals with right hemisphere dysfunction show deficits in music processing, affect, and pragmatic language skills [44]. Current intervention recommendations for individuals with auditory processing prosodic deficits often focus on keyword extraction and music therapy [44]. Since a focused, daily music curriculum has been found to be related to better pitch discrimination in typically developing children, further research should aim to examine these effects in children with known auditory processing disorders.

In an educational setting, pitch discrimination abilities have been linked to positive advances in phonological processing and indirectly to reading comprehension skills [25]. Additionally, because improvements in pitch discrimination ability have been linked to improvements in higher-level skills, such as phonological awareness and reading [9,25], it is possible that long-term music training may lead to further improvements in other academic skills. While further research is needed to show a causal effect, the present results speak to the potential benefit of focused, daily music training as a regular part of the elementary educational curriculum. 

## 5. Conclusions

In conclusion, children receiving more focused, daily musical training, even shown within a small sample size, show better scores for pitch discrimination abilities. Although statistical significance was not reached, a trend can be seen towards better performance for temporal sequencing and certain subtests of prosodic awareness (contrastive stress). This study holds important preliminary implications for the use of a focused, daily music curriculum in educational settings, as findings suggest that this type of music curriculum is associated with auditory processes that are ultimately associated with improved educational outcomes.

## Figures and Tables

**Table 1:** DLF scores (in Hz) and percent correct scores for each of the seven experimental conditions.

Participant	DLF	PPT	DPT	Turn-End	Affect	Boundary	CS
School 1							
1	4.91	47.22	76.67	100	94	50	75
2	9.48	86.67	66.67	100	88	75	88
3	5.58	100.00	33.00	100	88	88	100
4	3.34	83.33	66.67	100	63	88	81
5	3.23	50.00	56.67	100	94	94	100
6	9.59	80.65	40.00	94	94	75	81
7	4.91	66.67	40.00	100	81	75	50
8	21.20	46.67	66.67	94	75	88	81
School 2							
9	50.22	33.33	16.67	63	81	75	63
10	15.84	33.33	40.00	100	88	81	63
11	41.52	26.67	26.67	100	94	94	75
12	4.91	86.67	63.33	100	81	94	81
13	15.73	26.67	33.33	94	81	75	75
14	11.16	90.00	80.00	94	81	81	75
15	22.09	66.67	60.00	100	56	100	94
16	12.72	70.00	46.67	100	88	81	56

DLF: Difference limen for frequency; PPT: pitch pattern test; DPT: duration pattern test; CS: contrastive stress.

**Table 2:** Means and standard deviations per group for each experimental condition.

	School 1		School 2	
	Mean	SD	Mean	SD
DLF	7.78	5.95	21.77	15.81
PPT	70.15	20.50	54.16	27.07
DPT	55.79	16.08	45.83	20.99
Turn-end	98.50	2.78	93.88	12.77
Affect	84.63	11.08	81.25	11.29
Boundary	79.12	13.92	85.13	9.52
Contrastive Stress	82.00	15.86	72.75	11.99

## Data Availability

Individual data for each task can be found in Table 1 within the document.
